# Association of tumor size in pathological T4 colorectal cancer with desmoplastic reaction and prognosis

**DOI:** 10.1002/ags3.12571

**Published:** 2022-03-21

**Authors:** Takuya Shiraishi, Hiroomi Ogawa, Ayaka Katayama, Katsuya Osone, Takuhisa Okada, Yasuaki Enokida, Tetsunari Oyama, Makoto Sohda, Ken Shirabe, Hiroshi Saeki

**Affiliations:** ^1^ Department of General Surgical Science Gunma University Graduate School of Medicine Maebashi Japan; ^2^ Department of Diagnostic Pathology Gunma University Graduate School of Medicine Maebashi Japan

**Keywords:** colorectal cancer, desmoplastic reaction, epithelial‐mesenchymal transition‐associated histology, postoperative oncological prognosis, tumor size

## Abstract

**Background:**

Tumor size in pathological T4 (pT4) colorectal cancer (CRC) is associated with oncological prognosis; however, its relation to epithelial‐mesenchymal transition (EMT)‐associated histology is unclear. We aimed to investigate the association of tumor size with oncological prognosis and EMT.

**Methods:**

We performed a retrospective analysis of 95 patients with primary CRC who underwent radical surgery and were consecutively diagnosed with pT4.

**Results:**

Both 3‐y disease‐free survival (DFS) and cancer‐specific survival (CSS) were significantly higher in patients with tumor size ≥50 mm than in those with tumor size <50 mm (*P* = .009 and *P* = .011, respectively). The independent factors identified in the multivariate analysis for DFS were pathological lymph node metastasis (hazard ratio [HR], 2.551; 95% confidence interval [CI], 1.031–6.315; *P* = .043), distant metastasis (HR, 2.511; 95% CI, 1.140–5.532; *P* = .022), tumor size (HR, 0.462; 95% CI, 0.234–0.913; *P* = .026), and adjuvant chemotherapy (HR, 0.357; 95% CI, 0.166–0.766; *P* = .008). The independent factors identified in multivariate analysis for CSS were tumor location (HR, 10.867; 95% CI, 2.539–45.518; *P* = .001) and tumor size (HR, 0.067; 95% CI, 0.014–0.321; *P* < .001). In pT4 CRC, smaller tumor size was associated with nonmature desmoplastic reaction and EMT‐related histology.

**Conclusions:**

Tumor size ≥50 mm was associated with a better DFS and CSS than that of <50 mm, in patients with pT4 CRC. Smaller tumor size with advanced invasion likely reflects a more biologically aggressive phenotype in pT4 CRC.

## Graphical abstract

This retrospective study found a relationship between tumor size in colorectal cancer and epithelial‐mesenchymal transition‐related histology. In addition, this study found associations of tumor size with disease‐free survival and cancer‐specific survival.

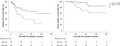

## 1 INTRODUCTION

In colorectal cancer (CRC), smaller tumor size has been reported to be associated with good survival and oncological prognosis.^1^, ^2^ Complete resection of smaller tumors could easily achieve remission compared to that of larger tumors, which have a higher risk of developing lymph node or distant metastasis.^3^ However, several recent studies reported that tumor size was not associated with survival,^4^, ^5^ while others showed that a smaller size had poor survival.^6^, ^7^ Furthermore, smaller tumors did not show superior survival compared to medium‐sized or larger tumors in patients with operable CRC.^5^ In patients with stage IIC, smaller tumor size was also associated with poorer survival than larger tumor size.^7^ Therefore, based on these recent findings, the association between tumor size and oncological prognosis is controversial; hence, no clear conclusions have been reached in CRC.

Cancer invasion and metastasis are affected by various stromal cells in the tumor microenvironment, as manifested by various pathological reactions.^8^ Promotion of epithelial‐mesenchymal transition (EMT), which allows epithelial cells to acquire the ability to invade and disseminate, can be morphologically identified on resected tissue specimens. In previous studies, EMT‐associated histology was evaluated using pathological desmoplastic reaction (DR) and poorly differentiated clusters (PDCs),^9^ and was found to be correlated with metastasis and worse oncological prognosis regardless of the CRC stage.^10^ DR is morphologically categorized on the basis of keloid‐like collagen and myxoid stroma, which are histological features that are closely related to the function of cancer‐associated fibroblasts (CAFs) that play a main role in mediating the EMT program in the cancer microenvironment.^10^, ^11^ PDCs are defined as clusters of five or more tumor cells without gland formation and characterize the migratory phenotype of a tumor.^12^ Therefore, DR and PDCs are thought to be morphological features of the tumor stroma that indicate EMT induction.^10^, ^13^

The pathological T4 (pT4) represents the most advanced tumor stage that is accompanied by serosa penetration (pT4a) and invasion of adjacent tissues/organs (pT4b), according to the TNM 8th edition.^14^ In clinical experience, pT4 CRC tumors can be identified either as large and localized without metastases or as small but highly invasive. Such tumors may have oncologically different phenotypes in pT4 CRC. Tumor size in pT4 CRC is associated with oncological prognosis and may potentially be related to the morphological features.^15^ However, no prior studies have investigated the relationship between tumor size and EMT‐related histology evaluated by DR and PDCs. Therefore, this study aimed to evaluate the association of tumor size with oncological prognosis and EMT, evaluated based on DR and PDCs, in patients with pT4 CRC who underwent curative resection. We also aimed to determine the cutoff for tumor size based on survival, the EMT‐associated histology assessed using DR and PDCs, and to analyze associations between tumor size and oncological prognosis.

## 2 PATIENTS AND METHODS

### 2.1 Patients and study design

We retrospectively analyzed all patients who underwent CRC surgery and were consecutively diagnosed with pT4 from June 2013 to March 2020, at the Gunma University Hospital in Japan. All cases with primary CRC were identified. Exclusion criteria were as follows: (a) patients who underwent preoperative treatments including chemotherapy and radiotherapy, and (b) patients who did not undergo radical surgery involving sites of distant metastasis.

Data regarding patient characteristics (age, sex, body mass index [BMI], tumor location, pathological findings including TNM classification, harvested lymph nodes, tumor size, postoperative hospital stay, morbidity, and use of adjuvant chemotherapy) and the surgical characteristics (operation type [colectomy, high anterior resection, low anterior resection, inter‐sphincteric resection, abdominoperineal resection, or total pelvic exenteration], approach type [open or laparoscopy], operation time, and blood loss) were collected for the analysis from medical and surgical records. Tumor size was assessed according to the maximum tumor diameter, which was measured by a pathologist relying on resected specimens. The follow‐up for patients was continued until February 2021. The study protocol was approved by the Institutional Review Board of Gunma University Hospital (approval no. HS2021‐020). The requirement for informed consent was waived because the analysis was based on a retrospective record review.

### 2.2 Evaluations of DR, PDCs, and EMT

An experienced pathologist, who was blinded to the patients’ clinical history or outcomes, reviewed the primary tumors to evaluate pathological DR, PDCs, and EMT. Hematoxylin and eosin‐stained glass slides of longitudinal sections of the deepest part of the tumor were microscopically scanned to evaluate DR and PDCs. Moreover, DR and PDCs were confirmed by evaluation by another pathologist who independently scored 50% of the cohort. The weighted κ values were good for grading DR and PDCs (*κ* = 0.8 and 0.7, respectively). Each parameter was evaluated according to the criteria provided in previous reports.^10^, ^12^, ^16^ DR was histologically classified using the following three categories: mature, intermediate, and immature DR.^16^ The evaluation was based on the existence of keloid‐like collagen and myxoid stroma, and the stroma was classified according to the most immature stromal area. Mature DR was diagnosed when fibrotic stroma was composed of fine, mature collagen fibers and did not contain keloid‐like collagen or myxoid stroma. Intermediate DR was diagnosed when keloid‐like collagen was present with mature stroma. Immature DR was diagnosed when the stroma with myxoid changes was present. PDCs were defined as clusters of five or more cancer cells infiltrating the stroma and lacking gland formation.^12^ Tumors with ≤4, 5–9, or ≥10 clusters were classified as G1, G2, or G3, respectively. EMT‐associated histology was based on DR and PDCs, and classified using three categories.^10^ Category A included tumors with both mature DR and G1 PDCs, category C included tumors with both immature DR and G3 PDCs, and category B included tumors with other types of DR and PDCs.

### 2.3 Postoperative treatment and follow‐up

We generally selected 5‐fluorouracil‐based chemotherapy for a total of 6 mo as postoperative adjuvant chemotherapy. At postoperative follow‐up, blood tests, including measurement of tumor markers, were performed at 3 mo, and enhanced abdominal and chest computed tomography scan was conducted every 6 mo. Colonoscopy was also performed every 1–2 y. In cases of suspected recurrence, positron emission tomography was performed, and the postoperative recurrences were confirmed clinically, histologically, or by consecutive radiologic follow‐up.

### 2.4 Statistical analysis

Quantitative variables are expressed as median and range. Categorical variables are expressed as frequency and percentage. The receiver‐operating characteristic (ROC) curve was used to determine the tumor size that optimally predicted postoperative recurrences. Comparison of tumor sizes was performed using Fisher's exact test or the chi‐square test. Univariate and multivariate analyses of risk factors for disease‐free survival (DFS) and cancer‐specific survival (CSS) using the Cox regression model were conducted. The multivariate analysis was performed using logistic regression with the backward stepwise method. All factors with *P* < .10 in the univariate analyses were included in multivariate analyses for DFS and CSS. The results of the Cox regression model are reported as hazard ratio (HR) and 95% confidence interval (CI). The 3‐y DFS (3y‐DFS) and the 3‐y CSS (3y‐CSS) were estimated using the Kaplan–Meier method, and differences were assessed using the log‐rank test. All statistical analyses were performed using SPSS (v. 27.0; IBM, Armonk, NY, USA), with the level of statistical significance set at *P* < .05.

## 3 RESULTS

### 3.1 Patient characteristics

During the study period, from June 2013 to March 2020, 140 patients underwent CRC surgery and were diagnosed with pT4. Of these, we excluded 15 patients who underwent preoperative treatments including chemotherapy and radiotherapy, and 30 who did not undergo radical surgery involving sites of distant metastasis. Finally, this study included 95 patients who underwent radical surgery involving sites of distant metastasis and were consecutively diagnosed with pT4 for primary CRC.

Patient characteristics are shown in Table 1. Of the 95 patients, the median age was 68 (range, 33–89) y; 53 were men (55.8%) and 42 women (44.2%); the median BMI was 21.3 (range, 14.5–33.4) kg/m^2^. In terms of tumor location, the cecum was the location in 10 cases (10.5%), ascending colon in 18 cases (18.9%), transverse colon in five cases (5.3%), descending colon in one case (1.1%), sigmoid colon in 29 cases (30.5%), and rectum in 32 cases (33.7%). Open and laparoscopic approaches were performed in 46 (48.4%) and 49 (51.6%) patients, respectively. pT4a and pT4b were diagnosed in 49 (51.6%) and 46 (48.4%) patients, respectively. The median maximum tumor size was 55.0 (range, 11.0–135.0) mm. A positive resection margin was present in 15 cases (15.8%). Adjuvant chemotherapy was administered in 61 patients (64.2%).

**TABLE 1:** Patient characteristics and pathological findings

	N = 95
Age (y), median (range)	68 (33–89)
Sex, N (%)	
Male	53 (55.8)
Female	42 (44.2)
Body mass index (kg/m^2^), median (range)	21.3 (14.5–33.4)
Tumor location, N (%)	
Cecum	10 (10.5)
Ascending	18 (18.9)
Transverse	5 (5.3)
Descending	1 (1.1)
Sigmoid	29 (30.5)
Rectum	32 (33.7)
Operative procedure, N (%)	
Colectomy	55 (57.9)
HAR	8 (8.4)
LAR	14 (14.7)
ISR	1 (1.1)
Hartmann	3 (3.2)
APR	6 (6.3)
TPE	8 (8.4)
Approach type, N (%)	
Open	46 (48.4)
Laparoscopy	49 (51.6)
Operative time (min), median (range)	311.0 (82.0–765.0)
Blood loss (mL), median (range)	145.0 (0.0–5507.0)
Morbidity (Clavien–Dindo all grade), N (%)	34 (35.8)
Depth of invasion (pT), N (%)	
pT4a	49 (51.6)
pT4b	46 (48.4)
Lymph node metastasis (pN), N (%)	
pN0	34 (35.8)
pN1	35 (36.8)
pN2	26 (27.4)
Distant metastasis (cM), N (%)	
cM0	77 (81.1)
cM1	18 (18.9)
Harvested lymph nodes, median (range)	26 (2–85)
Tumor differentiation, N (%)	
Well‐ or moderately‐differentiated tumor	49 (51.6)
Poorly‐differentiated tumor	46 (48.4)
Tumor maximum diameter (mm), median (range)	55.0 (11.0–135.0)
Radical resection margin, N (%)	
Negative	80 (84.2)
Positive	15 (15.8)
Adjuvant chemotherapy, N (%)	
Absence	34 (35.8)
Presence	61 (64.2)

Abbreviations: APR, abdominoperineal resection; HAR, high anterior resection; ISR, inter‐sphincteric resection; LAR, low anterior resection; TPE, total pelvic exenteration.

### 3.2 Relationship between tumor size and postoperative recurrences and the cutoff value

**FIGURE 1:**
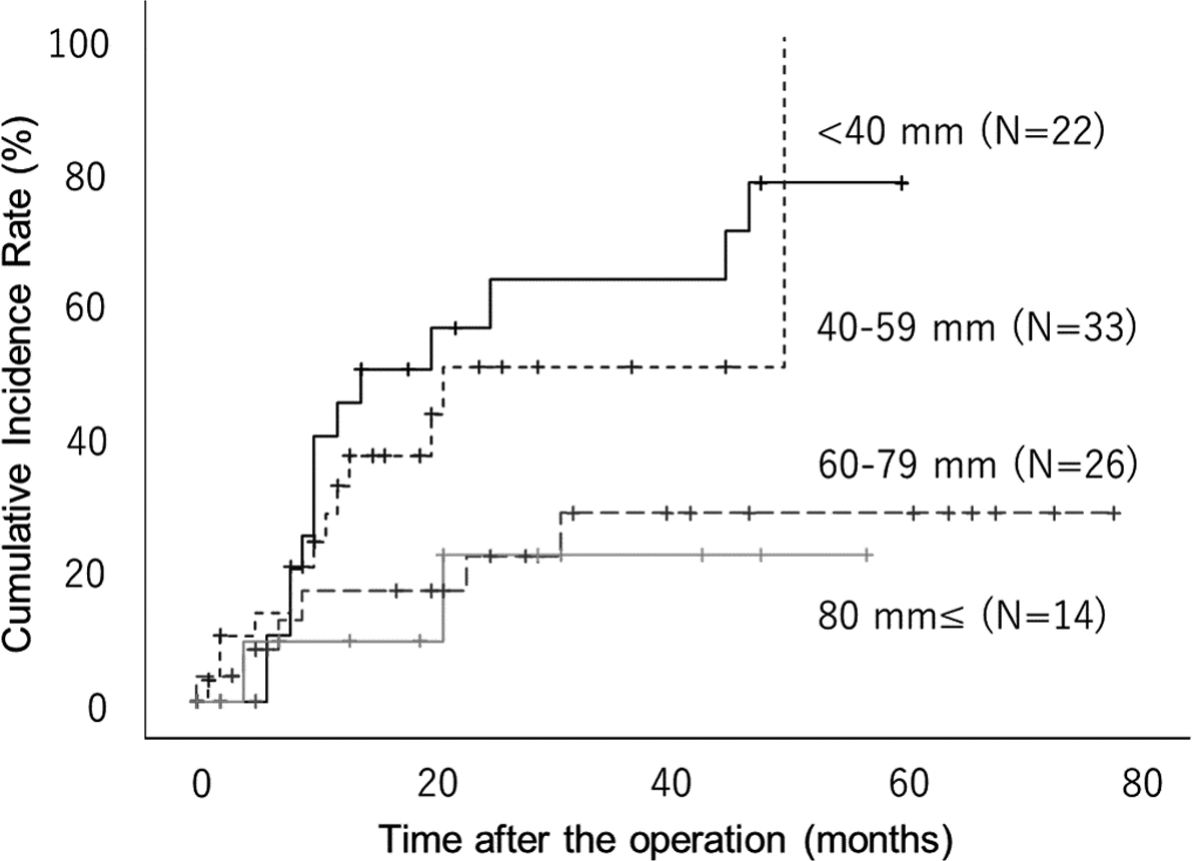
Association of postoperative recurrences with tumor size compared using cumulative incidence rate. The smaller the tumor size, the higher the rate of recurrence; the larger the tumor size, the lower the rate of recurrence (*P* = .012)

The association between postoperative recurrences and tumor size, compared using the cumulative incidence rate, is shown in Figure 1. The smaller the tumor was, the higher the rate of recurrence; the larger the tumor was, the lower the rate of recurrence (*P* = .012).

**FIGURE 2:**
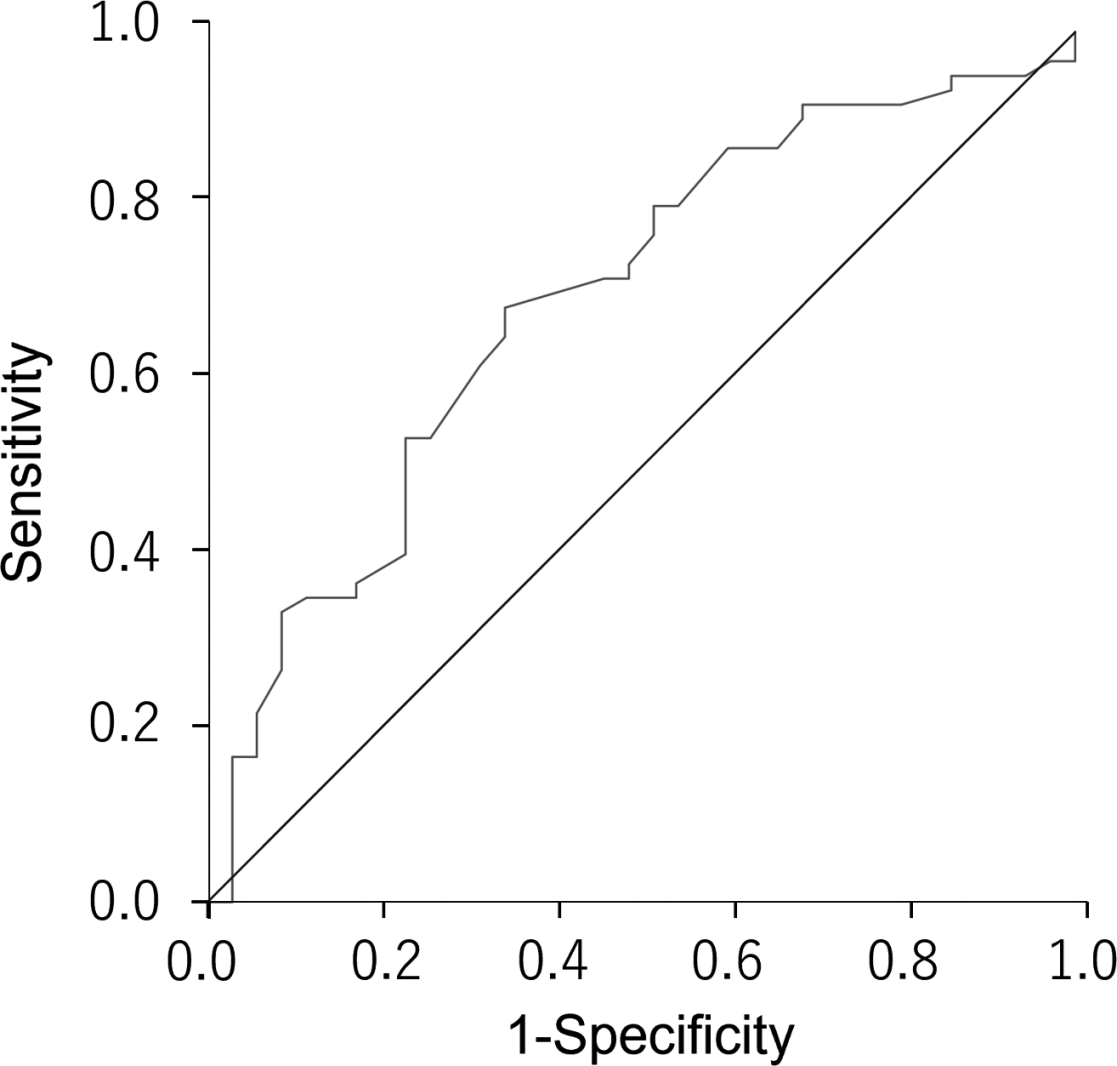
Prediction of recurrences after operation according to the tumor size. The area under the curve (AUC) for the tumor size is 0.682 (95% CI, 0.570–0.795). A cutoff value of 50 mm is selected, which maximizes specificity and sensitivity (66.7% and 65.7%, respectively), to predict the development of postoperative recurrences based on the tumor size

The ROC curve was used to determine the cutoff value for the tumor size that optimally predicted the development of postoperative recurrences. A cutoff value of 50 mm was selected, which maximized specificity and sensitivity (66.7% and 65.7%, respectively), for predicting postoperative recurrence based on tumor size (Figure 2). The area under the curve (AUC) for the tumor size was 0.682 (95% CI, 0.570–0.795).

Tumor size <50 mm was detected in 36 cases (37.9%), and ≥50 mm was detected in 59 cases (62.1%). Comparison of clinicopathological characteristics between patients with tumors <50 and ≥50 mm is shown in Table 2. A significant difference between these groups was found in the pathological T stage, while no statistically significant difference was found for other factors. A proportion of pT4b was significantly higher in the group of patients with tumors ≥50 mm than in those with tumors <50 mm (*P* = .002).

**TABLE 2:** Comparison of clinicopathological characteristics between the tumors <50 mm and ≥50 mm

	Tumor size <50 mm, N = 36	Tumor size ≥50 mm, N = 59	*P*
Age (y)			
<65	15 (41.7)	30 (50.8)	0.214
≥65	21 (58.3)	29 (49.2)	
Sex			
Male	23 (63.9)	30 (50.8)	0.351
Female	13 (36.1)	29 (49.2)	
Body mass index (kg/m^2^)			
<22	19 (52.8)	38 (64.4)	0.162
≥22	17 (47.2)	21 (35.6)	
Tumor location			
Colon	27 (75.0)	36 (61.0)	0.162
Rectum	9 (25.0)	23 (39.0)	
Approach type			
Open	13 (36.1)	33 (55.9)	0.061
Laparoscopy	23 (63.9)	26 (44.1)	
Operative time (min)			
<360	25 (69.4)	32 (54.2)	0.142
≥360	11 (30.6)	27 (45.8)	
Blood loss (mL)			
<100	20 (55.6)	21 (35.6)	0.057
≥100	16 (44.4)	38 (64.4)	
Morbidity (Clavien–Dindo grade ≥III)			
Absence	35 (97.2)	50 (84.7)	0.051
Presence	1 (2.8)	9 (15.3)	
Pathological T stage			
pT4a	26 (72.2)	23 (39.0)	0.002
pT4b	10 (27.8)	36 (61.0)	
Pathological lymph node metastasis			
Absence	10 (27.8)	24 (40.7)	0.203
Presence	26 (72.2)	35 (59.3)	
Distant metastasis			
Absence	28 (77.8)	49 (83.1)	0.525
Presence	8 (22.2)	10 (16.9)	
Harvested lymph nodes			
<12	1 (2.8)	3 (5.1)	0.511
≥12	35 (97.2)	56 (94.9)	
Tumor differentiation			
Well‐ or moderately‐differentiated tumor	14 (38.9)	32 (54.2)	0.146
Poorly‐differentiated tumor	22 (61.1)	27 (45.8)	
Radical resection margin			
Negative	30 (83.3)	50 (84.7)	0.855
Positive	6 (16.7)	9 (15.3)	
Adjuvant chemotherapy			
Absence	14 (38.9)	20 (33.9)	0.623
Presence	22 (61.1)	39 (66.1)	

### 3.3 Prognosis according to tumor diameter

**FIGURE 3:**
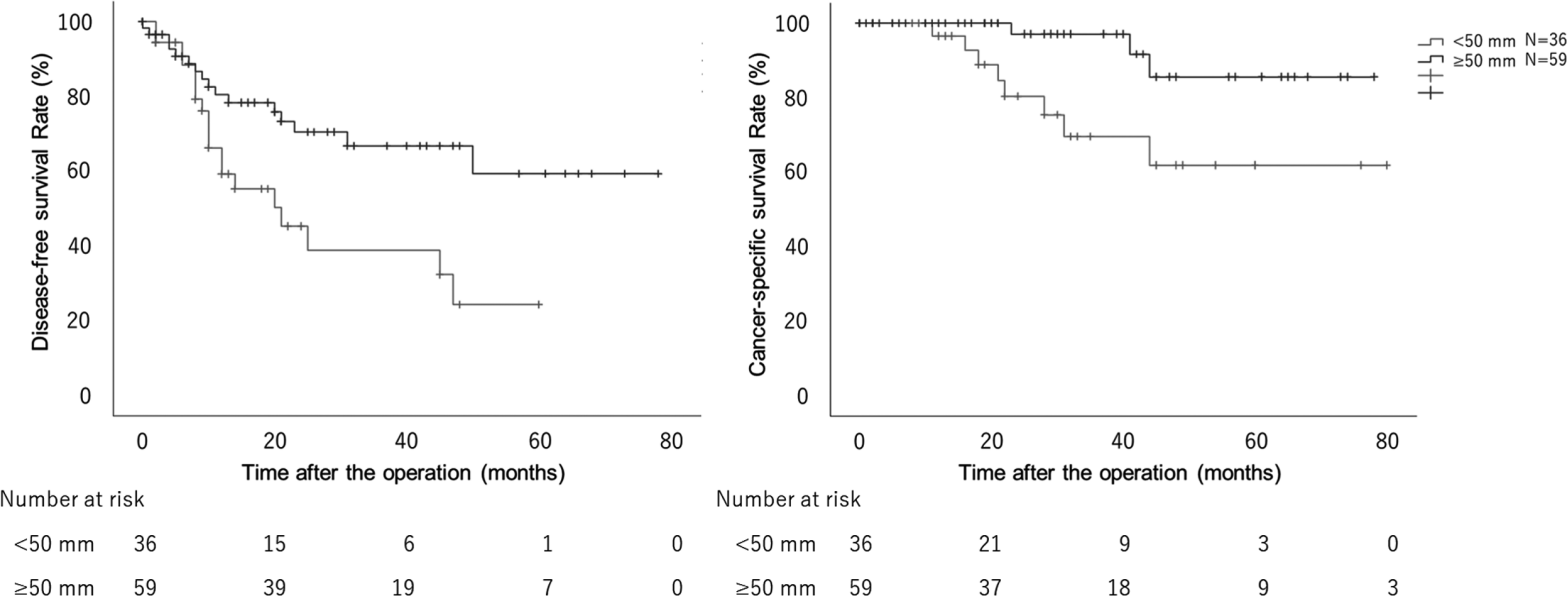
Disease‐free survival (DFS) and cancer‐specific survival (CSS) according to the tumor size. A, Kaplan–Meier curves for DFS according to the tumor size. The 3‐y DFS in patients with tumor size ≥50 mm is 66.7% and with that <50 mm is 38.7% (*P* = .009). B, Kaplan–Meier curves for CSS according to the tumor size. The 3‐y CSS in patients with tumor size ≥50 mm is 97.1% and with that <50 mm is 69.5% (*P* = .011)

The overall 3y‐DFS and 3y‐CSS were 56.7 and 86.2%, respectively. Figure 3 shows the Kaplan–Meier curves for DFS and CSS in patients with tumor size ≥50 mm and those with tumor size <50 mm. The 3y‐DFS was 66.7% in patients with tumors ≥50 mm and 38.7% in those with tumors <50 mm (*P* = .009). The 3y‐CSS was 97.1% in patients with tumors ≥50 mm and 69.5% in those with tumors <50 mm (*P* = .011).

Patients with pT4 were stratified with pT4a and pT4b, and the effect of tumor size in each stratum was assessed. In 49 patients with pT4a, the 3y‐DFS was 63.5% in patients with tumors ≥50 mm and 49.0% in those with tumors <50 mm (*P* = .299). The 3y‐CSS was 100.0% in patients with tumors ≥50 mm and 77.2% in those with tumors <50 mm (*P* = .135). The analysis of pT4a patients alone did not show any significant difference; however, prognosis tended to be poor for patients with tumors <50 mm compared to those with tumors ≥50 mm. In 46 patients with pT4b, the 3y‐DFS was 68.7% in patients with tumors ≥50 mm and 15.0% in those with tumors <50 mm (*P* = .003). The 3y‐CSS was 95.2% in patients with tumors ≥50 mm and 35.7% in those with tumors <50 mm (*P* = .002). In pT4b patients alone, the prognosis was significantly poor for patients with tumors <50 mm compared to those with tumors ≥50 mm.

### 3.4 Independent prognostic factors

Table 3 shows the univariate and multivariate analyses of the risk factors for DFS using the Cox regression model. Univariate analysis revealed that DFS was associated with pathological lymph node metastasis (*P* = .017), distant metastasis (*P* < .001), tumor differentiation (*P* = .016), tumor size (*P* = .012), and adjuvant chemotherapy (*P* = .026). The independent factors identified in the multivariate analysis were pathological lymph node metastasis (HR, 2.551; 95% CI, 1.031–6.315; *P* = .043), distant metastasis (HR, 2.511; 95% CI, 1.140–5.532; *P* = .022), tumor size (HR, 0.462; 95% CI, 0.234–0.913; *P* = .026), and adjuvant chemotherapy (HR, 0.357; 95% CI, 0.166–0.766; *P* = .008).

**TABLE 3:** Univariate and multivariate analysis of risk factors for disease‐free survival using the Cox regression model

Variable	Univariate analysis			Multivariate analysis		
	HR	95% CI	*P*	HR	95% CI	*P*
Age (y)						
<65	1		.943			
≥65	1.025	0.516–2.037				
Sex						
Male	1		.868			
Female	0.945	0.482–1.852				
Body mass index (kg/m^2^)						
<22	1		.353			
≥22	0.721	0.361–1.439				
Tumor location						
Colon	1		.179			
Rectum	1.584	0.809–3.102				
Approach type						
Open	1		.419			
Laparoscopy	1.319	0.674–2.581				
Operative time (min)						
<360	1		.590			
≥360	0.83	0.422–1.634				
Blood loss (mL)						
<100	1		.685			
≥100	0.871	0.448–1.696				
Morbidity (Clavien–Dindo grade ≥III)						
Absence	1		.759			
Presence	0.83	0.254–2.718				
Pathological T stage						
pT4a	1		.852			
pT4b	0.938	0.479–1.838				
Pathological lymph node metastasis						
Absence	1		.017	1		.043
Presence	2.754	1.201–6.312		2.551	1.031–6.315	
Distant metastasis						
Absence	1		<.001	1		.022
Presence	3.792	1.817–7.915		2.511	1.140–5.532	
Harvested lymph nodes						
<12	1		.683			
≥12	0.742	0.178–3.105				
Tumor differentiation						
Well‐ or moderately‐ differentiated tumor	1		.016			
Poorly‐differentiated tumor	2.416	1.180–4.947				
Tumor size (mm)						
<50	1		.012	1		.026
≥50	0.423	0.216–0.828		0.462	0.234–0.913	
Radical resection margin						
Negative	1		.328			
Positive	1.487	0.671–3.297				
Adjuvant chemotherapy						
Absence	1		.026	1		.008
Presence	0.445	0.218–0.909		0.357	0.166–0.766	

Abbreviations: CI, confidence interval; HR, hazard ratio.

Table 4 shows the univariate and multivariate analyses of risk factors for CSS using the Cox regression model. Univariate analysis revealed that CSS was associated with tumor size (*P* = .022). The independent factors identified by multivariate analysis were tumor location (HR, 10.867; 95% CI, 2.539–45.518; *P* = .001) and tumor size (HR, 0.067; 95% CI, 0.014–0.321; *P* < .001).

**TABLE 4:** Univariate and multivariate analysis of risk factors for cancer‐specific survival using the Cox regression model

Variable	Univariate analysis			Multivariate analysis		
	HR	95% CI	*P*	HR	95% CI	*P*
Age (y)						
<65	1					
≥65	0.887	0.269–2.920	.843			
Sex						
Male	1					
Female	0.670	0.196–2.289	.523			
Body mass index (kg/m^2^)						
<22	1					
≥22	0.492	0.130–1.856	.295			
Tumor location						
Colon	1			1		
Rectum	3.171	0.927–10.844	.066	10.867	2.539–45.518	.001
Approach type						
Open	1					
Laparoscopy	0.883	0.269–2.903	.838			
Operative time (min)						
<360	1					
≥360	1.349	0.411–4.428	.622			
Blood loss (mL)						
<100	1					
≥100	3.164	0.683–14.655	.141			
Morbidity (Clavien–Dindo grade ≥III)						
Absence	1					
Presence	0.669	0.085–5.265	.703			
Pathological T stage						
pT4a	1					
pT4b	1.151	0.347–3.813	.818			
Pathological lymph node metastasis						
Absence	1					
Presence	0.991	0.290–3.391	.989			
Distant metastasis						
Absence	1					
Presence	3.231	0.930–11.225	.065			
Harvested lymph nodes						
<12	1					
≥12	0.48	0.061–3.776	.485			
Tumor differentiation						
Well‐ or moderately‐differentiated tumor	1					
Poorly‐differentiated tumor	3.473	0.903–13.361	.070			
Tumor size (mm)						
<50	1			1		
≥50	0.211	0.056–0.798	.022	0.067	0.014–0.321	<.001
Radical resection margin						
Negative	1					
Positive	1.256	0.331–4.768	.737			
Adjuvant chemotherapy						
Absence	1					
Presence	1.092	0.222–5.363	.914			

Abbreviations: CI, confidence interval; HR, hazard ratio.

### 3.5 Relation between EMT‐related pathological factors and tumor size

The relation between EMT‐related histology and tumor size is shown in Table 5. Among the 59 patients with tumor size ≥50 mm, mature, intermediate, and immature DR were detected in 33 (55.9%), 19 (32.2%), and 7 (11.9%), respectively. Among the 36 patients with tumor size <50 mm, mature, intermediate, and immature DR were observed in eight (22.2%), 18 (50.0%), and 10 (27.8%), respectively. Mature DR was significantly more abundant in patients with tumor size ≥50 mm than in those with tumor size <50 mm (*P* = .004). Although the presence of PDCs did not show a significant association with tumor size (*P* = .128), EMT‐related histology was significantly associated with tumor size (*P* = .033). In pT4 CRC, smaller tumor size was associated with nonmature stroma and EMT‐related histology.

**TABLE 5:** Relation between EMT‐related pathological factors and tumor size

	Tumor size <50 mm, N = 36	Tumor size ≥50 mm, N = 59	*P*
DR			
Mature	8 (22.2)	33 (55.9)	.004
Intermediate	18 (50.0)	19 (32.2)	
Immature	10 (27.8)	7 (11.9)	
PDCs			
G1	17 (47.2)	33 (55.9)	.128
G2	8 (22.2)	18 (30.5)	
G3	11 (30.6)	8 (13.6)	
EMT‐related histology			
Category A	5 (13.9)	23 (39.0)	.033
Category B	29 (80.6)	34 (57.6)	
Category C	2 (5.6)	2 (3.4)	

Abbreviations: DR, desmoplastic reaction; EMT, epithelial‐mesenchymal transition; PDCs, poorly differentiated cluster.

### 3.6 Prognosis according to EMT‐related pathological factors

In DR, the 3y‐DFS was 57.6% in patients with matured DR, 60.6% in patients with intermediate DR, and 46.9% in those with immature DR (*P* = .642). The 3y‐CSS was 87.8% in patients with matured DR, 89.8% in patients with intermediate DR, and 77.9% in those with immature DR (*P* = .190). In PDCs, the 3y‐DFS was 65.5% in patients with G1 PDCs, 53.6% in patients with G2 PDCs, and 43.7% in those with G3 PDCs (*P* = .554). The 3y‐CSS was 86.6% in patients with G1 PDCs, 86.4% in patients with G2 PDCs, and 83.4% in those with G3 PDCs (*P* = .782). In EMT‐related histology, the 3y‐DFS was 71.5% in patients with category A, 52.2% in patients with category B, and 50.0% in those with category C (*P* = .258). The 3y‐CSS was 78.4% in patients with category A, 86.6% in patients with category B, and 50.0% in those with category C (*P* = .508).

## 4 DISCUSSION

We found that patients with tumor size ≥50 mm had better oncological prognosis than those with tumor size <50 mm in pT4 CRC. This result suggested that long‐term survival may be expected by aiming for R0 resection, representing the pathological complete resection, even if the tumor is large and resection of other organs is needed. Moreover, smaller tumor size with advanced invasion likely reflects a more biologically aggressive phenotype in pT4 CRC. Tumor size correlated significantly with postoperative oncological prognosis; specifically, tumor size <50 mm significantly correlated with poor DFS and CSS. pT4 cases with tumor size <50 mm were associated with nonmature DR and also with EMT‐related histology. In other words, a tumor that has acquired an invasive phenotype at an early stage and is characterized by smaller tumor size may be prone to recurrence or metastasis and lead to poor survival.

In the present study, larger tumor size was associated with better survival than smaller tumor size in pT4 CRC. Several studies have reported similar results.^7^ Huang et al^7^ also suggested an association between increased tumor size and superior CSS and identified 50 mm as an optimal cutoff value, which was the same as that in our study. Moreover, they reported that, compared with larger tumor size, smaller tumor size (<50 mm) was associated with poorer CSS in a large cohort of stage II colon cancer (pT3/pT4 and pN0). Our results provided new knowledge that a cutoff value of 50 mm was also adapted for locally advanced cancer, pT4, with lymph node metastasis. Furthermore, after pT4 was stratified into pT4a and pT4b, the relationship between tumor size and prognosis was analyzed in pT4a and pT4b; the relationship between tumor size and prognosis was greater for tumors invading other organs (pT4b). Tumors with a larger size and no distant metastasis progress only locally; therefore, radical surgical resection may contribute to long‐term survival in these patients even if the tumor is large with T4 category, especially tumors invading other organs. From these results, the locally advanced large CRC may have a unique biological behavior with a lower propensity to metastasize.

Previous studies have shown that DR is associated with the EMT of neoplastic cells and that nonmature DR is a characteristic of aggressive tumors.^10^, ^17^ The cases with nonmature DR showed adverse clinicopathological findings in CRC, such as advanced T stage and poor oncological prognosis. The association of nonmature DR and EMT in pT4 cases with tumor size <50 mm suggests that there might be some underlying molecular mechanisms causing the poor oncological prognosis. Some previous studies have shown that the microenvironment induced by CAFs is regulated by various growth factors secreted by cancer cells that support cancer invasion and metastasis; also, the heterogeneity of the CAFs may be manifested by the differential DR patterns.^13^, ^18^ Nonmature DR correlated with the morphology of CAFs that promotes invasion and metastasis of cancer cells.^19^ Therefore, tumors with nonmature DR that gained an invasion ability at an early stage, characterized by smaller tumor size, may be at a high risk for metastasis and poor survival. In the Sakura trial,^17^ DR was a poor oncological prognostic factor independent of the stage, and nonmature DR was the reason for recommending additional treatments, such as postoperative chemotherapy for stage II patients. As shown by the Sakura trial, more aggressive treatments should probably be selected for the pT4 patients with smaller tumor size and nonmature DR to improve postoperative oncological prognosis. Although PDCs were reported to be associated with EMT and oncological prognosis along with DR,^12^ there was no association between PDCs and tumor size in the present study. Because PDCs were evaluated at the invasive tumor front, and it may be difficult to evaluate PDCs at the serosal invasion or other organ invasion, the association between PDCs and tumor size was not found in the present study. In contrast, larger tumors were not associated with DR and EMT‐related histology. If the tumors remain localized and have not acquired metastatic potential, even though they are large and pT4, these patients with a locally advanced tumor may be cured by surgery alone.

The difficulty of surgery and a possible need for resection of other organs are higher when the tumor size is large, and as a result there is an increase in the incidence of postoperative complications and a decrease in quality of life when other organs are resected.^20^ Nevertheless, our results suggest that in the case of large tumors, a better long‐term survival may be expected than that with small tumors with R0 resection, even when resection of other organs is needed. Indeed, one of the main factors associated with long‐term survival for CRC is R0 resection.^21^, ^22^ Achieving R0 resection often requires a high standard of surgical skill, especially when combined with resection of other organs, which is the case for T4 tumors. In addition, compared to standard surgery, the incidence of postoperative complications is increased in other organ resections, such as pelvic exenteration.^20^, ^23^ Therefore, it is very important to determine whether R0 resection can be definitely achieved before surgery, because it is sometimes burdensome for patients, although T4 CRC patients with large tumors are likely to have long‐term survival if R0 resection is achieved. On the other hand, small tumors that invade other organs may have very aggressive properties because this group has poor prognosis even if R0 resection is achieved; therefore, these patients may require multidisciplinary treatment before radical surgery to improve the postoperative oncological prognosis.

In addition to tumor size, pathological lymph node metastasis, distant metastasis, and adjuvant chemotherapy were associated with DFS, and tumor location was associated with CSS. The rectum has unique anatomic and physiologic features, which increase the risk of local spread and postoperative recurrence of rectal cancer compared to colon cancer. Therefore, as shown in our result, rectal cancer is reported to be associated with worse postoperative survival compared to colon cancer.^24^ The presence of pathological lymph nodes metastasis is an important prognostic factor in CRC. Lymph node metastasis cause higher recurrence rates and shorter survival, making adjuvant chemotherapy important in improving the prognosis for these CRC patients with lymph node metastasis.^22^, ^25^ The goal of adjuvant chemotherapy is eradication of clinically occult micrometastases to increase the cure rate after a potentially curative resection for CRC patients. The benefits of adjuvant chemotherapy have been most clearly demonstrated in stage III CRC; it is now a standard treatment strategy in the guidelines of the Japanese Society for Cancer of the Colon and Rectum.^22^, ^25^, ^26^ Also in stage II CRC, pT4 targeted in this study is a risk factor for postoperative recurrence, and adjuvant chemotherapy is recommended in the guidelines, including the American Society of Clinical Oncology and European Society for Medical Oncology. Recently, pT4 was also classified as a higher‐risk group of postoperative recurrence in the International Duration of Adjuvant Chemotherapy collaboration; therefore, the presence or absence of adjuvant chemotherapy was an important factor related to postoperative recurrence.^27^, ^28^ The presence of distant metastasis from colorectal cancer does not contraindicate curative treatment, unlike many other cancers. Achieving complete resection for CRC patients, even with distant metastasis, could possibly cure. However, the postoperative recurrence rate is high, especially following resection of synchronous metastasis.^29^ Remarkably, tumor size was an independent prognostic factor even including these previous prognostic factors; it is thought that the patients with smaller tumor sizes and a strong invasive tendency have very aggressive cancers.

This study had several limitations. First, it must be acknowledged that the study design was retrospective in nature and that it included a small sample size of CRC cases from a single institution. Because of the small sample size, no significant results were found to show that the EMT‐histological findings were associated with an unfavorable prognostic factor in CRC patients, although a similar tendency was found in our study as in previous reports.^9^, ^10^ Second, although we compared the pathological results with tumor size, which was associated with postoperative oncological prognosis, we have not conducted molecular analyses in the present study. Hence, multicenter, large‐scale prospective studies are needed to further confirm our results, and they should also tackle the molecular context of these tumors. Third, it might be biased to include patients with synchronous distant metastasis, even those achieved by curative resection. However, we analyzed patients, excluding those with synchronous distant metastasis, and the results were similar to the present results. Despite these limitations, we believe our results provide clinically useful information. First, our study suggests that the small T4 tumors have acquired an early invasion ability and their oncological prognosis is poor; therefore, more aggressive treatments may be needed. Second, our study highlights that it is important to perform radical resection, because patients with large T4 tumors are likely to have long‐term survival if R0 resection is achieved.

In conclusion, we found that tumor size significantly correlated with postoperative survival. Specifically, tumor size <50 mm significantly correlated with poor DFS and CSS. pT4 cases with tumor size <50 mm were associated with nonmature DR and also EMT‐related histology. In these cases, patients may require multidisciplinary treatment before radical surgery to improve the postoperative oncological prognosis. Additionally, our results suggest that the timing of acquisition of metastatic and invasion ability may be associated with the oncological prognosis in CRC.

## ETHICS APPROVAL

The study protocol was approved by the Institutional Review Board of Gunma University Hospital (approval no. HS2021‐020). The requirement for informed consent was waived because the analysis was based on a retrospective record review.

## DISCLOSURE

Funding: The authors have not received financial support from any funding sources for this study.

Conflict of Interest: Author KS is an editorial board member of Annals of Gastroenterological Surgery. The authors declare no conflicts of interest for this article.

Author Contribution**:** TS collected data, wrote the article, and prepared the figures. AK evaluated data. HO, KO, TO, YE, TO, MS, KS, and HS revised the article and provided comments on the structure and details of the article. All authors read and approved the final article.
